# Calculating Strain Energy Release Rate, Stress Intensity Factor and Crack Propagation of an FGM Plate by Finite Element Method Based on Energy Methods

**DOI:** 10.3390/ma18122698

**Published:** 2025-06-08

**Authors:** Huu-Dien Nguyen, Shyh-Chour Huang

**Affiliations:** 1 Faculty of Technology, Long An University of Economics and Industry, No.938, QL1 Rd, Khanh Hau Ward, Tan An 82113, Vietnam; nguyen.dien@daihoclongan.edu.vn; 2 Department of Mechanical Engineering, National Kaohsiung University of Science and Technology, No.415, Jiangong Rd, Sanmin Dist, Kaohsiung 807618, Taiwan

**Keywords:** SIFs, FEM, FGM, J-integral, theory of minimum strain energy density, theory of maximum energy release, theory of maximum tangential normal stress

## Abstract

In the field of crack mechanics, predicting the direction of a crack is important because this will evaluate whether, when the crack propagates, it penetrates into important areas and whether the structure is dangerous or not. This paper will refer to three theories that predict the propagation direction of cracks: a theory of maximum tangential normal stress, a theory of maximum energy release, and a theory of minimum strain energy density. At the same time, the finite element method (FEM)–ANSYS program will be used to calculate stress intensity factors (SIFs), strain energy release rate (J-integral), stress field, displacement near a crack tip, and crack propagation phenomenon based on the above theories. The calculated results were compared with the results in other scientific papers and experimental results. This research used ANSYS program, an experimental method combined with FEM based on the above energy theories to simulate the J-integral, the SIFs, and the crack propagation. The errors of the SIFs of the FGM rectangular plate has a through-thickness center crack of 1.77%, J-integral of 4.49%, and crack propagation angle $\theta_{c}$ of 0.15%. The FEM gave good errors compared to experimental and exact methods.

## 1. Introduction

FGM is a new-generation composite which is applied in aeronautical engineering (engineering fuselage), in medicine (making teeth, artificial bones), in national defense (bulletproof armor), and in the energy industry (insulation panels, turbines, reactors). The FGM material is a combination of two materials in which the volume ratio of each component changes smoothly and continuously [1,2,3,4,5].

In recent years, FEM is a numerical method that is widely used in mechanics to predict and model the mechanical behavior of structural materials. The FEM has emerged as an effective technique for analyzing crack problems. It is increasingly being used as a viable method in crack modeling that develops under the assumption of linear elastic fracture mechanics [6,7,8].

Furthermore, the FEM is one of the most popular numerical tools for finding approximate solutions to differential equations and partial differential equations. It has been successfully applied in many fields of science and technology to study, simulate, and predict the behavior of structures. Simulating cracks in textures requires the FEM to mesh in accordance with the pore geometry. This method is computationally expensive and complicated and also generates less accurate results to problems when updating the old mesh to a newer version, especially when there are many cracks [9].

For certain layers of FGM, the stress and displacement of cracks asymptotically come from fields of the same form as those in a homogeneous material (Eischen, 1983 [5]). Characteristic variations in the effects of materials manifest in near-SIFs and in higher-order conditions in asymptotic expansion. Erdogan (1995) [6] wrote an excellent review article on this topic. Although several analytic expressions for the stress strength factor for cracks in the FGM have been derived, the investigation is limited to semi-infinite or infinite domains and simple load cases. Analytical expressions for mixed-mode SIFs were obtained recently by Gu and Asaro (1997) [9]. In this case, the crack tip is oriented perpendicular to the material gradient. Although an infinite domain, Nonda and Erdogan (1994) [10] obtained a more general case of material gradients related to crack orientation. In order to correlate the fracture strength data and determine the critical crack length in more general samples, it is clear that a numerical method is necessary to determine the mixed-mode SIF in the FGM [10,11].

Computational fracture mechanics has progressed significantly in last decades, with diverse new methods for the stress evaluation, allowing us to evaluate complex fracture mechanics problems with relatively low computational costs. For metallic structures, as well as aluminum aircraft structures, typical approaches are based on linear-elastic fracture mechanics to evaluate the fatigue crack propagation in order to compute the fatigue life of these structures under different loading and boundary conditions. The greatest computational cost in these analyses is the characterization of stress and displacements fields, required for the determination of stress intensity factors (SIFs) [7].

The SIF is an extremely important parameter in crack mechanics, indicating a degree of stress concentration at a crack tip. In three-dimensional space, the SIFs, $K_{I}$, $K_{II}$, and $K_{III}$, characterize the three independent displacements of a crack, including mode I, mode II, and mode III. When predicting the propagation direction of two-dimensional cracks, the three energy methods, $\sigma_{\theta \theta max}$, $S_{min}$, and $G_{max}$, all use two important parameters, $K_{I}$ and $K_{II}$, to calculate the bending angle of the crack [12,13,14].

This paper will present the theories based on these methods and some simple propagation crack models referenced from other references. We use ANSYS software 19.2 based on above three energy theories to calculate SIFs, strain energy release rate (J-integral), and crack propagation angle $\theta_{c}$ of a rectangular, isotropic FGM plate having a through-thickness center crack and an internal crack. These mechanisms were also employed to check the experimental and the exact methods of calculating the J-integral, SIFs, and crack propagation phenomenon of a rectangular, isotropic FGM plate.

## 2. Method to Calculate the Propagation Direction of the Crack

### 2.1. The Theory of Maximum Tangential Normal Stress $\sigma_{\theta \theta max}$

The mixed expressions of an elastic stress field around a crack tip when expressed in polar coordinates are as follows [15]:

(1) $$\sigma_{rr}=\frac{1}{\sqrt{2\pi r}}cos\frac{\theta }{2}\left[K_{I}\left(1+{sin}^{2}\frac{\theta }{2}\right)+\frac{3}{2}K_{II}sin\theta -2K_{II}tan\frac{\theta }{2}\right]$$

(2) $$\sigma_{\theta \theta }=\frac{1}{\sqrt{2\pi r}}cos\frac{\theta }{2}\left[K_{I}{cos}^{2}\frac{\theta }{2}-\frac{3}{2}K_{II}sin\theta \right]$$

(3) $$\sigma_{r\theta }=\frac{1}{2\sqrt{2\pi r}}cos\frac{\theta }{2}\left[K_{I}sin\theta +K_{II}\left(3cos\theta -1\right)\right]$$

In which, $K_{I}$ and $K_{II}$ are the two SIFs that characterize the two independent displacement forms of the crack, which are mode I and mode II.

**Figure 1:**
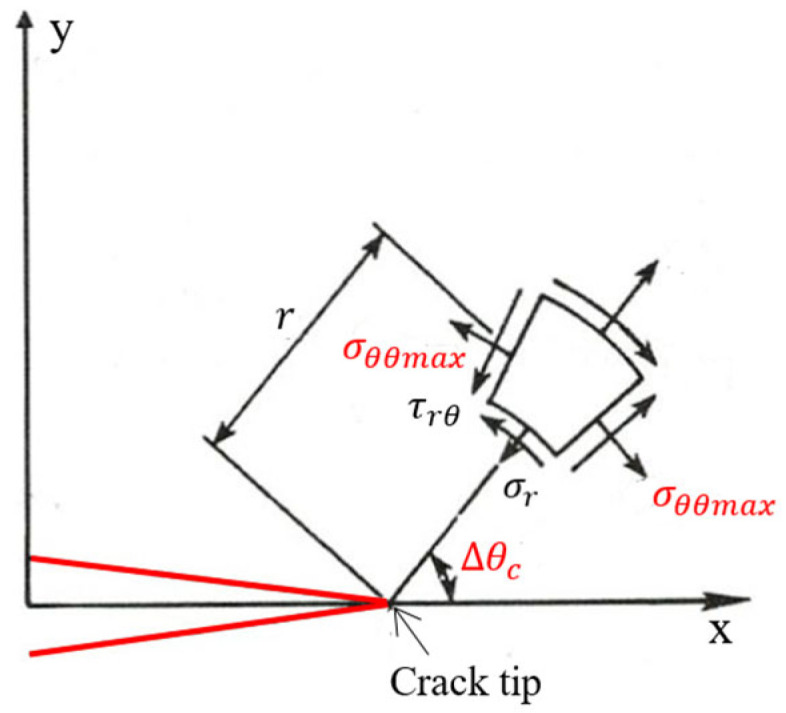
Maximum tangential normal stress in polar coordinate system.

The theory of maximum tangential normal stress $\sigma_{\theta \theta max}$ (first order) for materials confirms that a crack will develop in the direction perpendicular to the theory of maximum tangential normal stress. This theory was proposed by Sih and Erdogan in 1963 [15] (see Figure 1).

Derive Equation (2) in terms of the variable θ and assign it to 0.

(4) $$\frac{\partial \sigma_{\theta \theta }}{\partial \theta }=0$$

After rearranging and setting $\theta =∆\theta_{c}$, Equation (2) will have the following form:

(5) $$\frac{K_{II}}{K_{I}}=\frac{-\sin∆\theta_{c}}{3\cos∆\theta_{c}-1}$$

Solving Equation (5) according to the variable $∆\theta_{c}$, The bending angle of the crack will be calculated.

According to [16], based on the theory of maximum tangential normal stress, the bending angle of the crack can also be calculated from the following formula:

(6) $$∆\theta_{c}=\arccos\left[\frac{3K_{II}^{2}+\sqrt{K_{I}^{4}+8K_{I}^{2}K_{II}^{2}}}{K_{I}^{2}+9K_{II}^{2}}\right]$$

In addition, the bending angle of the crack can also be calculated according to the formula from reference [17] as follows:

(7) $$∆\theta_{c}=2\arctan\left[\frac{-2K_{II}/K_{I}}{1+\sqrt{1+8{\left(K_{II}/K_{I}\right)}^{2}}}\right]$$

According to Formula (7), if $K_{II}=0$, then $∆\theta_{c}=0$ (purely opening form (mode I only)). If $K_{II}>0$, then the crack angle $∆\theta_{c}<0$. If $K_{II}<0$, then the crack angle $∆\theta_{c}>0$.

### 2.2. The Theory of Minimum Strain Energy Density $S_{min}$

This theory was proposed by Sih in 1974 [18]. Sih developed the formula for calculating a strain energy density S according to stress intensity factors $K_{I}$ and $K_{II}$ as follows:

(8) $$S=a_{11}K_{I}^{2}+2a_{12}K_{I}K_{II}+a_{22}K_{II}^{2}$$

with

(9) $$a_{11}=\frac{1}{16\mu }\left[\left(1+cos\theta \right)\left(\kappa -cos\theta \right)\right]$$

(10) $$a_{12}=\frac{1}{16\mu }sin\theta \left[2cos\theta -\left(\kappa -1\right)\right]$$

(11) $$a_{22}=\frac{1}{16\mu }\left[\left(\kappa +1\right)\left(1-cos\theta \right)+\left(1+cos\theta \right)\left(3cos\theta -1\right)\right]$$

where E is elastic modulus, and ν is Poisson’s ratio; shear module $\mu =\frac{E}{2\left(1+\nu \right)}$; $\kappa =\frac{3-\nu }{1+\nu }$ for plane stress; κ=3−4ν for the plane strain.

A crack will grow in a direction $\theta =∆\theta_{c}$, where the strain energy density is minimal.

(12) $$\frac{dS}{d\theta }=0\;\mathrm{and}\;\frac{d^{2}S}{d\theta^{2}}>0$$

The crack starts to propagate when the strain energy density reaches its maximum value $S=S_{cr}$.

According to reference [19], the maximum value $S_{cr}$ is calculated according to the following formula:

(13) $$S_{cr}=\left(1-2\nu \right)\left(1+\nu \right)K_{IC}^{2}/2E$$

where $K_{IC}$ is the critical SIF.

### 2.3. Theory of Maximum Energy Release $G_{max}$

**Figure 2:**
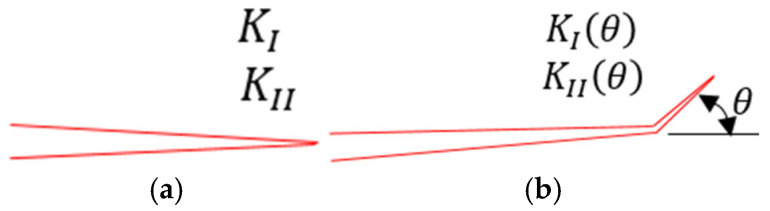
Initial primary crack with a bending part with angle θ: (**a**) original crack; (**b**) kinked crack.

This theory is based on the calculation of Hussain in 1974 [20]. These are the SIFs, $K_{I}(\theta )$ and $K_{II}(\theta )$, of an initial major crack with a bending part with a very small angle θ at the tip, calculated based on the SIFs, $K_{I}$ and $K_{II}$, of normal cracks (see Figure 2).

(14) $$K_{I}\left(\theta \right)=g\left(\theta \right)\left(K_{I}\cos\theta +\frac{3}{2}K_{II}\sin\theta \right)$$

(15) $$K_{II}\left(\theta \right)=g\left(\theta \right)\left(K_{II}\cos\theta -\frac{3}{2}K_{I}\sin\theta \right)$$

(16) $$g\left(\theta \right)=\left(\frac{4}{3+\cos^{2}\theta }\right){\left(\frac{1-\theta /\pi }{1+\theta /\pi }\right)}^{\frac{\theta }{2\pi }}$$

According to Irwin’s general expression [21], the energy release rate G for the initial crack with a bent part with an angle θ will be given as follows:

(17) $$G\left(\theta \right)=\frac{1}{E^{\prime }}\left(K_{I}^{2}\left(\theta \right)+K_{II}^{2}\left(\theta \right)\right)$$

with $E^{\prime }=E$ for plane stress, and $E^{\prime }=\frac{E}{\left(1-\nu^{2}\right)}$ for plane strain.

Combined with Equations (14)–(16), Equation (17) becomes the following:

(18) $$G\left(\theta \right)=\frac{1}{4E^{\prime }}g^{\prime }\left(\theta \right)\left[\left(1+3\cos^{2}\theta \right)K_{I}^{2}-8\sin\theta \cos\theta K_{I}K_{II}+\left(9-5\cos^{2}\theta \right)K_{II}^{2}\right]$$

The propagation angle of the crack is found by minimizing G(θ).

(19) $$\frac{\partial G\left(\theta \right)}{\partial \theta }=0$$

And the following stability conditions must be satisfied:

(20) $$\frac{\partial^{2}G\left(\theta \right)}{\partial \theta^{2}}<0$$

The general form of Equation (18) can be abbreviated as follows:

(21) $$G\left(\theta \right)=\frac{1}{4E^{\prime }}\left[A_{11}K_{I}^{2}\left(\theta \right)+A_{22}K_{II}^{2}\left(\theta \right)+2A_{12}K_{I}\left(\theta \right)K_{II}\left(\theta \right)\right]$$

(22) $$\left[\begin{array}{c} A_{11}\\ A_{12}\\ A_{22} \end{array}\right]=g^{2}\left(\theta \right)\left[\begin{array}{c} 4-3\sin^{2}\theta \\ -2\sin2\theta \\ 4+5\sin^{2}\theta \end{array}\right]$$

### 2.4. Comparison Between Three Methods of Calculating Crack Propagation Direction

The following is a formula comparing the results between the theory of maximum tangential normal stress with the theory of maximum energy release and the theory of minimum strain energy density as referenced from [22,23,24,25,26]. For the convenience of comparison, we put the following:

(23) $$M^{e}=\frac{2}{\pi }\tan^{-1}\left(\frac{K_{I}}{K_{II}}\right)$$

In addition, this research would like to present another comparison between the three methods of calculating the propagation direction of the crack. This result is referenced from the literature [15,18,20,27,28,29,30,31,32].

**Figure 3:**
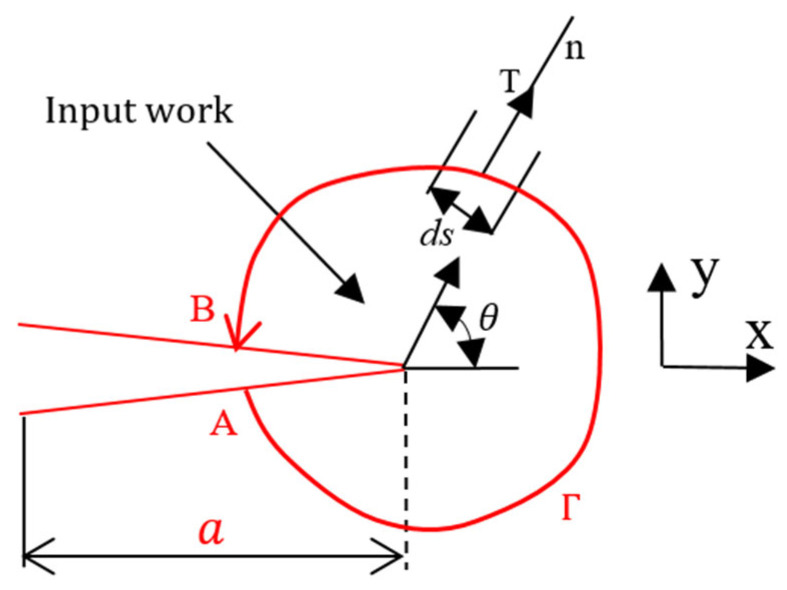
J-integral contours around the crack surfaces.

Rice [33] and Eshelby [34] introduced a contour integral surrounding a crack front, as illustrated in Figure 3:

(24) $$J=\int_{\Gamma }\left(Wdy-\vec{T}\frac{\partial \vec{\mu }}{\partial x}ds\right)$$

Here, *J* denotes the energy release rate (J/m^2^); *W* represents the elastic strain energy density (J/m^3^); $\vec{\mu }$ is the displacement vector evaluated at the differential element ds; *n* is the outward unit normal to the contour Γ; ds is a differential segment along the contour; $\vec{T}\left(\partial \vec{u}/\partial x\right)ds$ corresponds to the input work; s denotes the arc length; Γ is an arbitrary counterclockwise contour; $\vec{T}$ refers to the traction (tensile) vector acting on the body enclosed by Γ.

Rice [33] and Riveros [35] certified a path independent concepts and found that stress energy release rate G is equal to J-integral:

(25) $$J=G=\frac{K_{I}^{2}+K_{II}^{2}}{E^{\prime }}$$

where $E^{\prime }=E$ for plane stress, $E^{\prime }=\frac{E}{\left(1-\nu^{2}\right)}$ for plane strain.

## 3. A Results and Discussions: A FGM Plate with the Through-Thickness Center Crack

**Figure 4:**
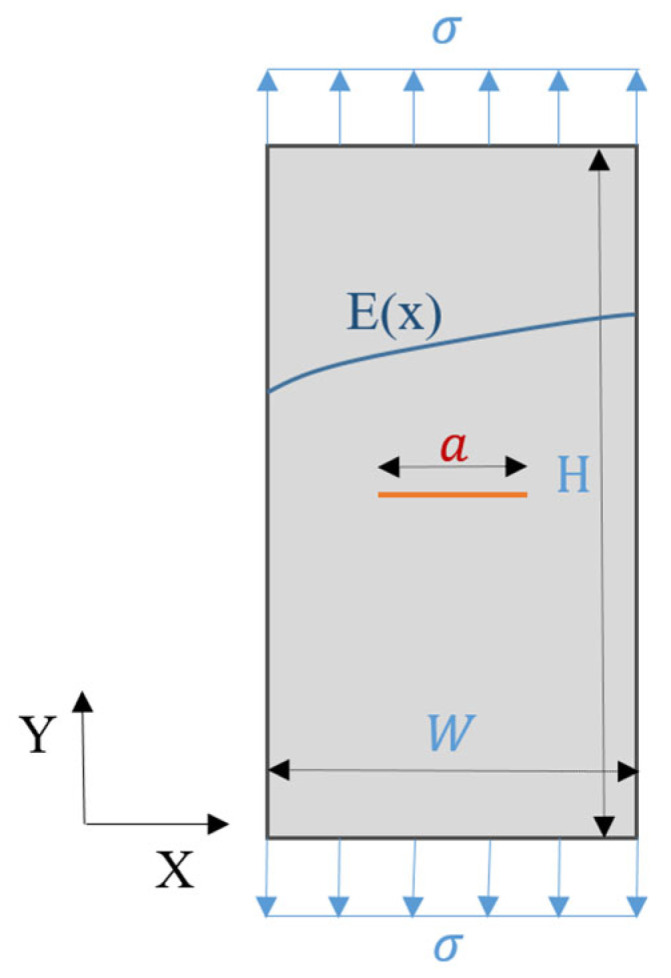
A through-thickness center crack model.

Figure 4 illustrates the FGM plate along with its boundary conditions and dimensions. The material was characterized by a Poisson’s ratio of ν = 0.25, with geometric parameters a = 0.5 cm, H = 10 cm, and W = 2 cm. An applied stress of *σ* = 10^6^ N/m^2^ was considered. The Young’s modulus *E* varied exponentially across the plate from the left to the right edge. The analysis was conducted under the assumption of a plane strain condition.

(26) $$E\left(x\right)=E_{0}e^{\lambda x}\;$$

where $E_{0}=10^{3}$ Pa, and λ=2.

**Figure 5:**
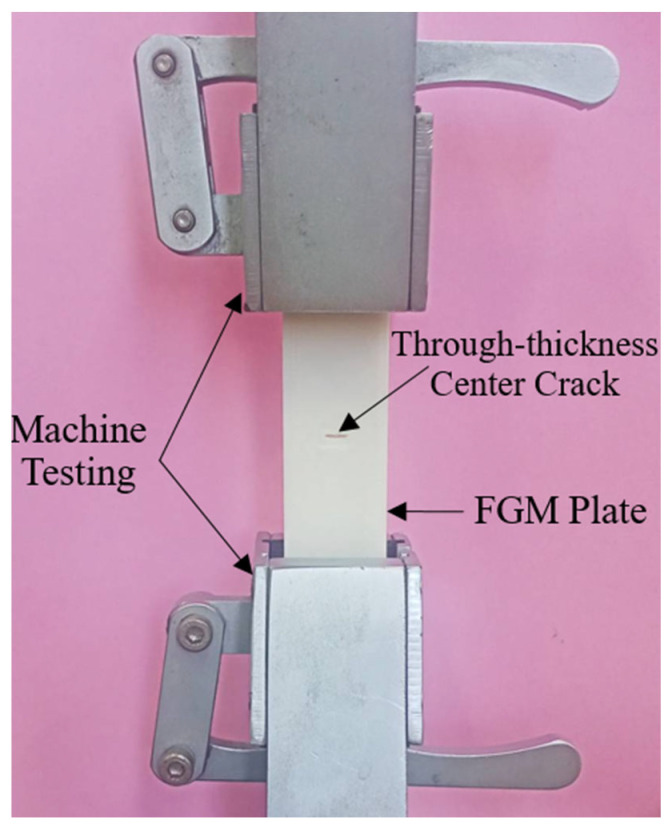
The experimental setup.

Experimental tests were performed on specimens with dimensions *H* = 10 cm, *W* = 2 cm, *a* = 0.5 cm, and a thickness of *t* = 0.5 cm, fabricated using 3D printing technology. The specimens were made of nylon ABS, with a Poisson’s ratio of *ν* = 0.25, and prepared in accordance with the ASTM E399 standard; ASTM International, West Conshohocken, PA, USA, 11 July 2023. Tests were conducted using an LF2373 Friction Tester under varying loading conditions. Two testing speeds were applied: v_1_ =2 mm/min for Case 1 and v_2_ =3 mm/min for Case 2. In both cases, a constant load of *F* = 100 N was applied, based on the relationship *F* = *σ*/*A*, where *A* = *W* × *t* is the cross-sectional area. The experimental setup is shown in Figure 5.

**Figure 6:**
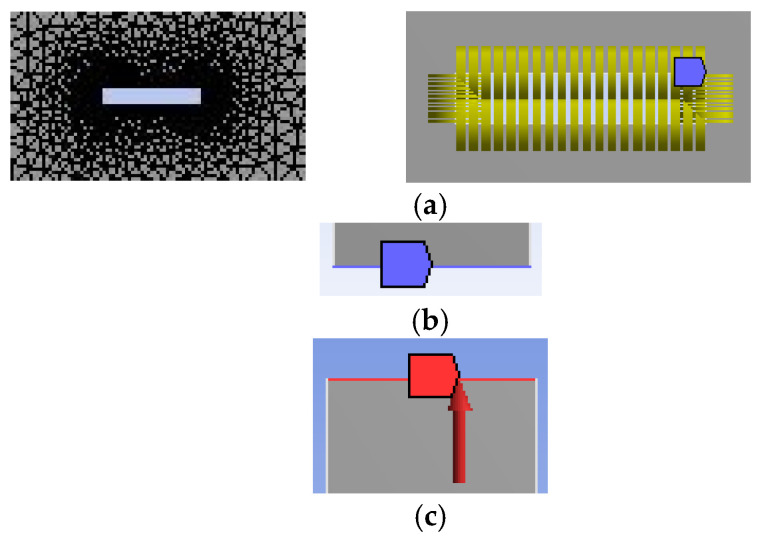

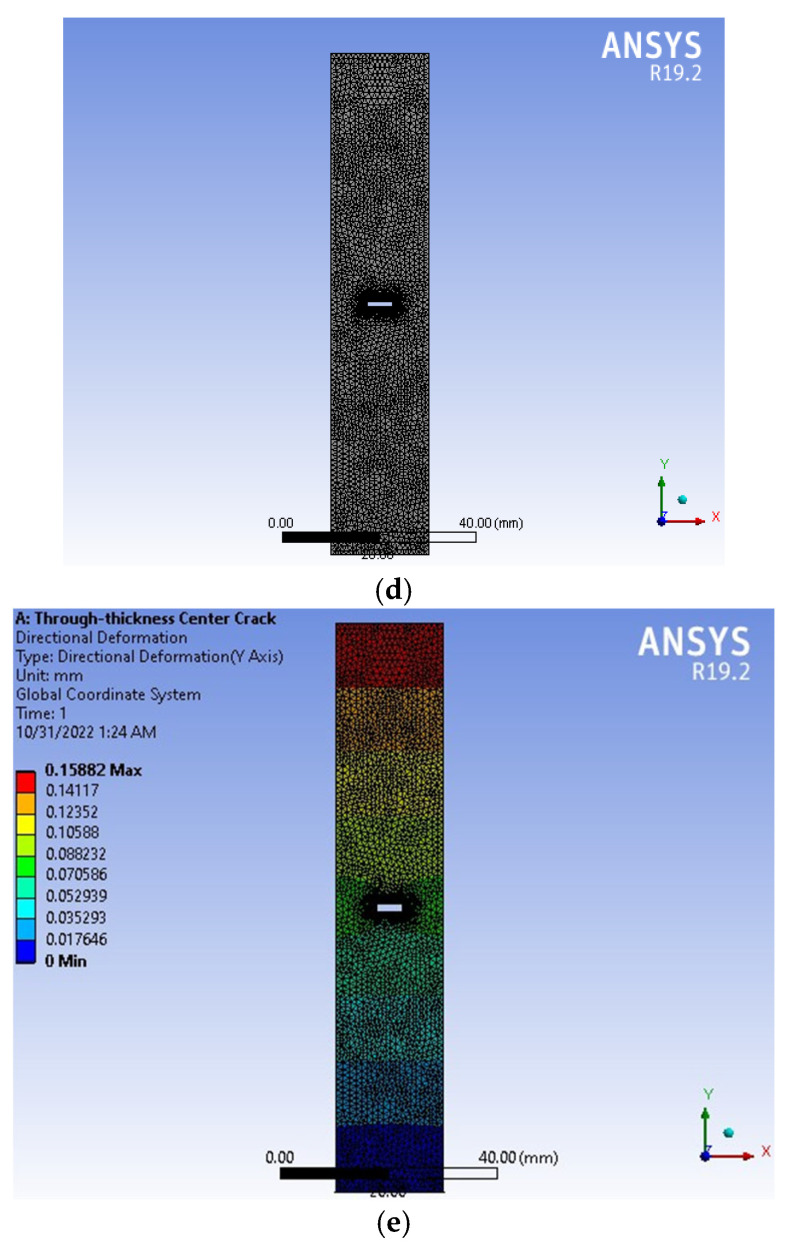
(**a**) The finite element mesh was refined in the vicinity of the crack. (**b**) The bottom edge of the specimen was fixed. (**c**) A uniform tensile displacement was applied at the top edge. (**d**) The general model used in the numerical analysis. (**e**) The results of displacement $u_{y}$.

**Figure 7:**
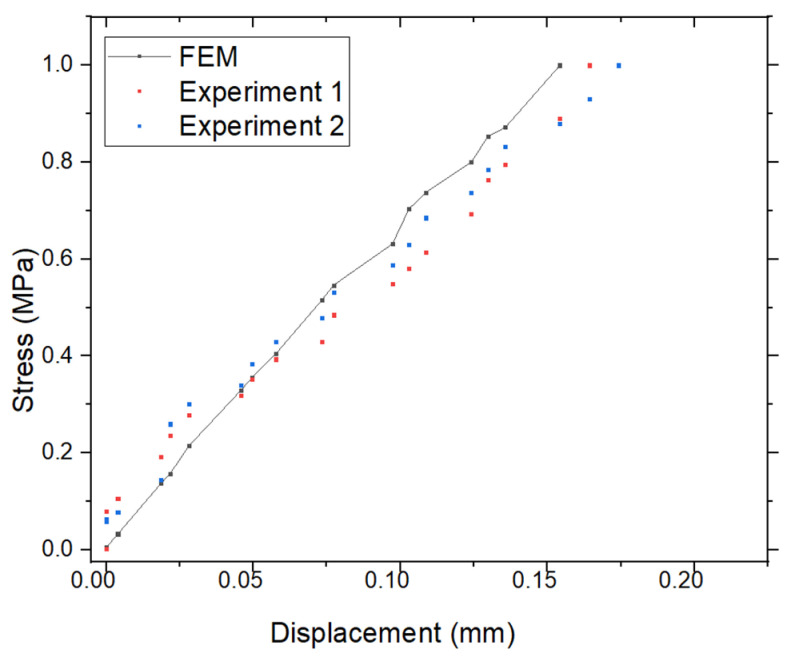
Comparison of displacement $u_{y}$ of FEM and experimental results.

The vertical displacements $u_{y}$ obtained from the finite element method (FEM) are presented in Figure 6, corresponding to the model with dimensions H = 10 cm, a = 0.5 cm, and W = 2 cm (see model in Figure 4). The maximum displacement predicted by FEM is Max($u_{y}$) = 0.15882 mm. In comparison, experimental measurements yielded Max($u_{y}$) = 0.16335 mm for Case 1, with a percentage error of 5.55%, and Max($u_{y}$) = 0.17032 mm for Case 2, corresponding to a percentage error of 6.42%. These results are summarized in Figure 6 and Figure 7 and Table 1. Figure 7 illustrates the stress–displacement curves obtained from the FEM and the two experimental cases. The maximum stress was observed to be Max($\sigma_{yy})=1$ MPa (or 10^6^ N/m^2^), with all three curves showing close agreement. These findings confirm that the FEM approach provides reliable predictions when validated against experimental data.

**Figure 8:**
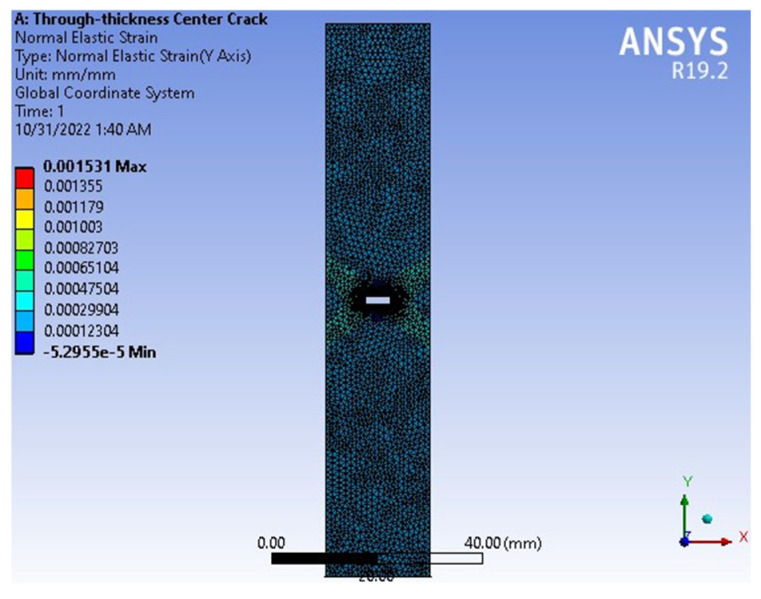
The results of strain $\varepsilon_{y}.$

**Figure 9:**
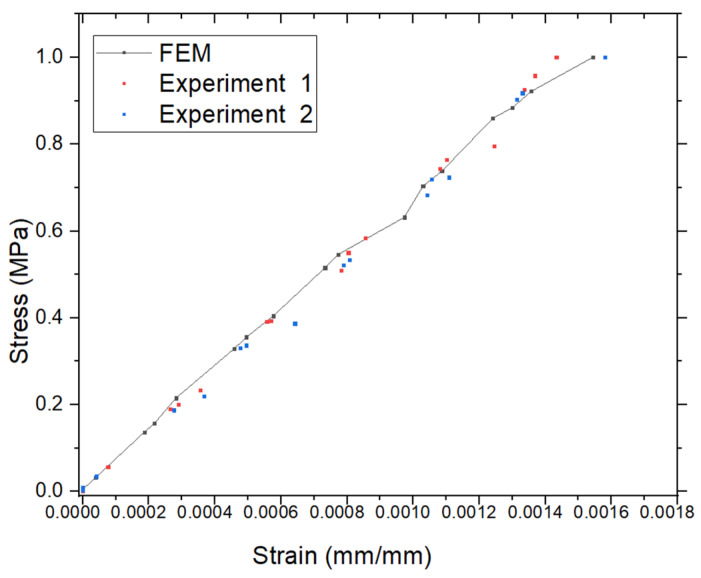
Comparison of strain $\varepsilon_{y}$ of FEM and experimental results.

The strain results $\varepsilon_{y}$, obtained using the finite element method (FEM), are shown in Figure 8 for a model with dimensions H = 10 cm, a = 0.5 cm, and W = 2 cm, consistent with the geometry presented in Figure 4. The maximum strain calculated by the FEM is Max($\varepsilon_{y}$) = 0.001531 = 0.001531 mm/mm. Experimental results yielded Max($\varepsilon_{y}$) = 0.0014833 mm/mm for Case 1, corresponding to a percentage error of 3.12%, and Max($\varepsilon_{y}$) = 0.001579 mm/mm for Case 2, with an error of 3.13%. These findings are summarized in Figure 8 and Figure 9 and Table 1. Figure 9 presents the stress–strain curves obtained from FEM and both experimental cases. The maximum stress was observed to be Max($\sigma_{yy})=1$ MPa (or 10^6^ N/m^2^), and the three curves show a high degree of consistency. These results further confirm the accuracy and reliability of the FEM in predicting mechanical behavior compared to experimental methods.

**Figure 10:**
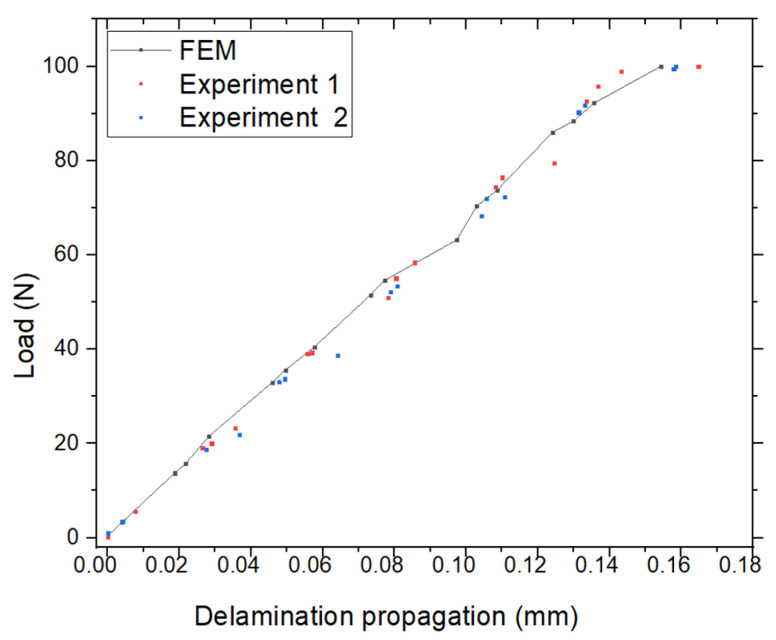
Comparison of delamination propagation along the *y*-direction between FEM simulation and experimental results.

Figure 10 presents the delamination propagation results along the *y*-direction, obtained from FEM simulations and two experimental cases. The maximum delamination predicted by the FEM is Max($\varepsilon_{y}$) = 0.1543 mm. In comparison, experimental results show Max($\varepsilon_{y}$) = 0.1648 mm for Case 1, with a percentage error of 6.37%, and Max($\varepsilon_{y}$) = 0.1585 mm for Case 2, with an error of 2.65%. The load–delamination propagation curves for FEM and both experimental cases are also illustrated in Figure 10. The maximum applied load was F = 100 N, calculated based on the relation F = σ/A, where σ = 10^6^ N/m^2^ and the cross-sectional area A = 2 × 0.5 × 10^−4^ m^2^. The three curves exhibit close agreement, confirming the consistency between the FEM predictions and experimental observations. These results further validate FEM as a dependable tool for analyzing delamination behavior.

**Figure 11:**
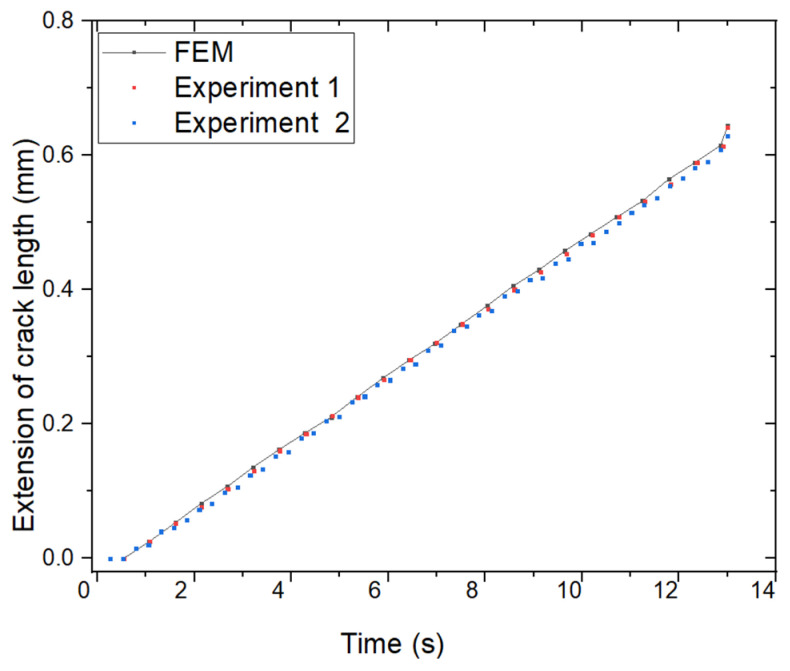
Crack length extension ∆a: FEM vs. experimental results.

Results of the FEM and two case experiments show that the extension of crack length ∆a of a FGM plate are illustrated in Figure 11. The maximum extension of the crack length by the FEM at the time t=13 (s) was Max(∆a) = 0.6439 (mm), by the experiment for Case 1 was Max(∆a) = 0.6423 mm, % Error = 0.25%; and for Case 2 was Max(∆a) = 0.6286 mm, % Error = 2.43%. Figure 11 illustrates the crack length extension versus time curves obtained from the FEM analysis and two experimental cases. The three curves exhibit nearly identical trends, indicating a strong correlation between numerical predictions and experimental observations. These findings further confirm the reliability and accuracy of the FEM approach in modeling crack propagation behavior.

As shown in the newly included Figure 6a, the finite element mesh was refined in the vicinity of the crack to capture the high stress gradients accurately. A mapped mesh was employed in the crack region with a higher element density, while a coarser mesh was used in regions far from the crack to optimize computational efficiency. The element type used was PLANE183 for 2D modeling, which is suitable for simulating linear elastic behavior in fracture mechanics.

The specimen was modeled with symmetry in both geometry and loading conditions. Displacement boundary conditions were applied as follows: the bottom edge of the specimen was fixed in the Y-direction (to prevent rigid body motion), and a uniform tensile displacement was applied at the top edge in the Y-direction. The lateral edges were left free to simulate uniaxial tensile loading. These settings replicate a mode I crack-opening scenario under plane stress conditions. These updates are now described in Figure 6b,c. The general model used in the numerical analysis, including crack geometry, boundary conditions, and material gradient direction, is presented in Figure 6d.

The stress intensity factor (SIF) analysis results, based on the model parameters *W* = 2 cm, *σ* = 10^6^ N/m^2^, and *a* = 0.5 cm, are taken from reference [30] as follows:

(27) $$K_{I}=\alpha \sigma \sqrt{\pi a}=4.2152\;\mathrm{MPa}\sqrt{mm}$$

The crack geometry correction factor is adopted from Ref. [30] as follows:

(28) $$\alpha \approx {\left[1+0.5{\left(a/w\right)}^{2}+20.46{\left(a/w\right)}^{4}+81.72{\left(a/w\right)}^{6}\right]}^{1/2}=\;1.0635$$

Based on Equation (25), the following results were obtained:

(29) $$E^{\prime }=\frac{E}{\left(1-\nu^{2}\right)}=\;1009.0817\;Pa$$

(30) $$J=\frac{K_{I}^{2}}{E^{\prime }}=0.0176\;\times \;10^{12}\;\mathrm{Pa}\cdot \mathrm{mm}=\;0.0176\mathrm{mJ}/{\mathrm{mm}}^{2}$$

**Figure 12:**
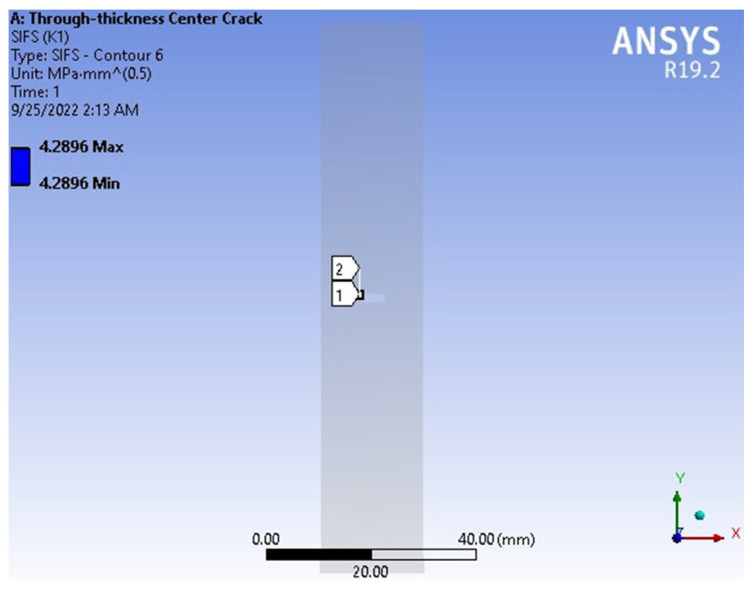
The result of SIF.

**Figure 13:**
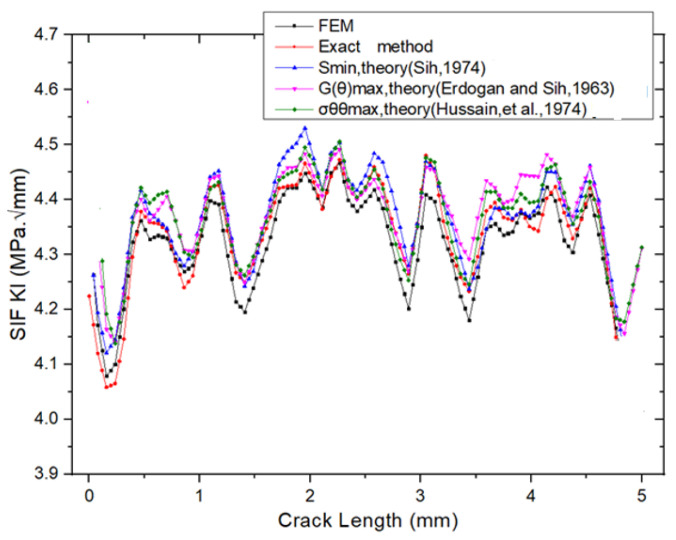
Comparison of SIFs versus crack length of FEM, exact, and other reference results (Sih, 1974 [14]; Erdogan and Sih, 1963 [16]; Hussain, et al., 1974 [20]).

The mode I stress intensity factors (SIFs), $K_{I}$, obtained from the FEM analysis are presented in Figure 12. The FEM yielded a value of $K_{I}$ = 4.2896 MPa·$\sqrt{mm}$, while the exact method produced $K_{I}$ = 4.2152 MPa·$\sqrt{mm}$, corresponding to a percentage error of 1.77%, as reported in Table 2. Figure 13 provides a comparison of SIF versus crack length curves obtained from FEM, the exact method, and other reference results. All five curves demonstrate close agreement. These findings confirm that FEM provides accurate and reliable predictions for stress intensity factors.

**Figure 14:**
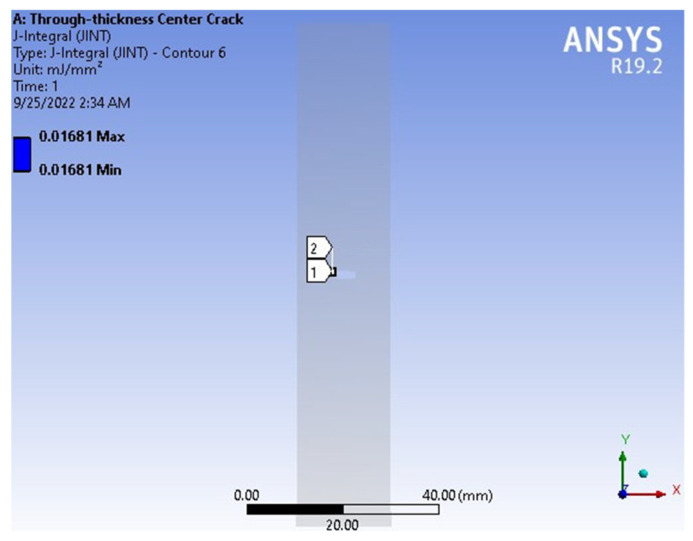
J-integral results obtained from FEM.

**Figure 15:**
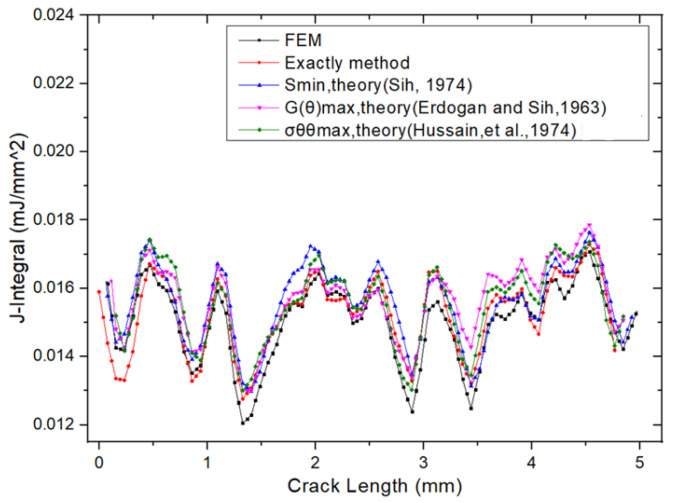
Comparison of J-integral versus crack length of FEM, exact, and other reference results (Sih, 1974 [14]; Erdogan and Sih, 1963 [16]; Hussain, et al., 1974 [20]).

Figure 14 presents the J-integral results obtained using the finite element method (FEM) and the exact method (as defined in Equation (30)). The FEM yielded a value of J = 0.01681 mJ/mm^2^, while the exact method produced J = 0.0176 mJ/mm^2^, resulting in a percentage error of 4.49%, as reported in Table 3. Figure 15 shows a comparison of the J-integral versus crack length curves obtained from FEM, the exact method, and other reference data. The five curves are in close agreement, further confirming that the FEM provides reliable and accurate results for fracture analysis.

**Figure 16:**
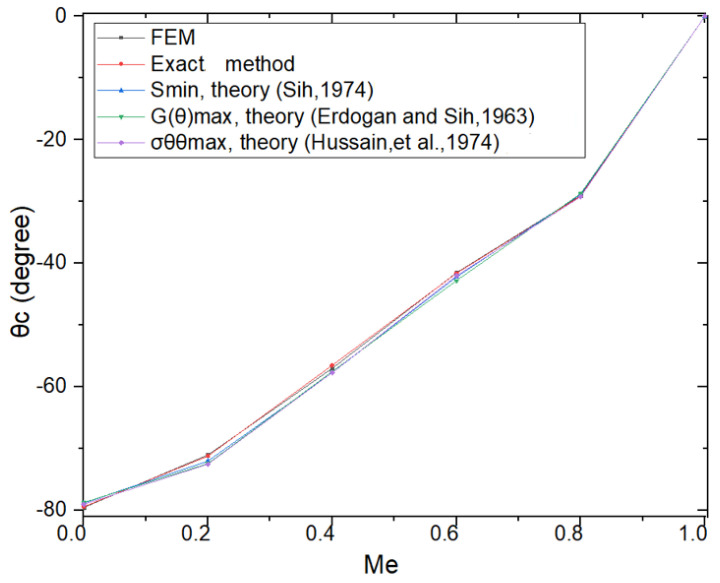
Comparison of modeling of $\theta_{c}$ versus $M^{e}$ of FEM, exact, and reference energy methods (Sih, 1974 [14]; Erdogan and Sih, 1963 [16]; Hussain, et al., 1974 [20]).

The results $M^{e}$ (see Equation (23)) and $\theta_{c}$ (see Equation (7)) are shown in Figure 16—by the FEM, exact, and reference energy methods. $M^{e}$ by the FEM: $M^{e}=$ 0.31, by the exact method: $M^{e}=$ 0.32; % Error $M^{e}$ by the FEM compared to the exact method: Error = 3.12%; Values $\theta_{c}$ by FEM: $\theta_{c}=$ −64.5°; by the exact method θ= −64.6°; % Error $\theta_{c}$ by the FEM compared to the exact method: Error =0.15% (Table 4). Because values $K_{II}>0$, values $\theta_{c}<0$—by theory (Equation (7)). Figure 16 compares the curves $\theta_{c}$ versus $M^{e}$ of the FEM, exact method, and reference energy methods; the five curves lie the same. The values $M^{e}$ calculated at each step according to the three theories are approximately equal to 1 (between 0–1). Therefore, the bending angle of the crack calculated according to the three theories at each step has nearly equal values. This is consistent with the graph comparing the results between the three theories that predict the propagation direction of the crack referenced from the literature. Therefore, the crack path modeled according to the three theories has almost same form. The results illustrate that the FEM is the reliable method.

The following criteria were compared: 

a. Maximum Tangential Stress (MTS) Criterion:

Advantage: Simple and widely used, especially for brittle materials; provides good agreement with experimental data under mode I-dominated loading. Disadvantage: Less accurate for mixed-mode loading or ductile fracture. Applicability: Effective in predicting initial crack propagation in brittle materials, such as ceramics or high-strength steels.

b. Maximum Energy Release Rate (G-max) Criterion:

Advantage: Energy-based and theoretically sound; considers both mode I and mode II contributions. Disadvantage: Requires accurate energy release rate calculation; less intuitive than stress-based methods. Applicability: Suitable for materials with significant energy dissipation, such as composites or polymers.

c. Minimum Strain Energy Density (SED) Criterion:

Advantage: Accounts for the energy density around the crack tip, providing a more localized perspective. Disadvantage: Highly sensitive to mesh refinement and may yield ambiguous directions in certain cases. Applicability: Useful in complex geometries or when stress fields are not symmetric.

The influence of these criteria on the predicted crack path and the observed deviations in the numerical results is also discussed. This comparative analysis helps identify the most suitable criterion for different material types and loading conditions in practical fracture mechanics applications.

A comparative analysis between our proposed method and other relevant studies in the field of crack mechanics and finite element simulation is provided. The proposed approach was compared with studies by Sih, 1974 [18], Erdogan and Sih, 1963 [19], and Hussain et al., 1974 [20], which utilize similar FEM-based techniques for crack propagation analyses. The comparison highlights the following key points of novelty and practical contribution in this work: 

1. Novelty: Unlike prior studies that primarily focus on stress intensity factor evaluation or static crack modeling, the proposed approach integrates dynamic crack propagation modeling, multi-theory comparison, and refined meshing near crack tips within the ANSYS environment, providing enhanced accuracy and broader applicability. 

2. Practical Application: The methodology demonstrated in this paper can be directly applied to structural integrity assessment in components such as pressure vessels, pipelines, and aircraft panels, where understanding crack direction and growth behavior is critical for failure prevention.

**Figure 17:**
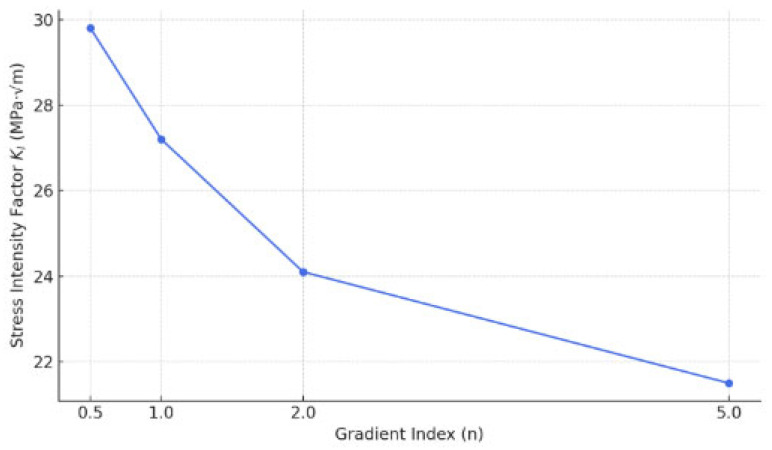
Effect of gradient index on SIF $K_{I}$ in FGM.

In the revised manuscript, we have included a new subsection (Section 3, lines 329–350), presenting a parametric study that evaluates the influence of different gradient indices (n = 0.5, 1, 2, and 5) on the stress intensity factors (SIFs) and crack propagation direction (Figure 17). The results demonstrate that a higher gradient index generally leads to a reduction in SIF values due to the increased contribution of the tougher material near the crack tip. Crack paths tend to curve toward the region with lower stiffness, consistent with previous observations in FGM fracture mechanics. Additionally, we briefly discuss how alternative material combinations (e.g., ceramic–metal vs. metal–metal FGMs) could further impact crack growth behavior, and we outline this as a direction for future work to keep the scope focused.

## 4. Conclusions

In this research, we use the FEM to simulate the crack tip behavior in two dimensions, and stress–displacement fields near a crack tip could be more exact compared with the experimental method. The calculation of the SIFs and J-integral will be faster than the analytical theory when encountering a complex crack model. Concurrently, it was possible to relatively calculate the growth and shape of the two-dimensional crack, as it propagates under different conditions of the model, material, and loading method.

The SIFs, J-integral, and crack propagation angle mentioned in Section 3 were calculated based on three energy methods. The results obtained via the FEM were better than those obtained using the experimental method, when compared to the exact method. Findings can be introduced as a novel approach to calculate the SIF, J-integral, and crack propagation phenomenon of the FGM plate.

Further, this research presented the FEM based on the three energy methods above to calculate the SIFs, J-integral, and crack propagation phenomenon of the FGM plate based on the reference models. Such mechanisms have widespread applications in industrial practice and real-world engineering.

## Figures and Tables

**Table 1:** Comparison of a maximum strain and displacement between FEM and experimental method.

	FEM	Exper.		Error (%)	
		Exper. 1	Exper. 2	Exper. 1	Exper. 2
Displacement $u_{y}$ (mm)	0.15882	0.16335	0.17032	5.55	6.42
Strain $\varepsilon_{y}$ (mm/mm)	0.001531	0.0014833	0.001579	3.12	3.13

**Table 2:** Comparison of SIFs $K_{I}$ between the exact method and the FEM.

Exact (MPa·$\sqrt{mm}$)	4.2152
FEM (MPa·$\sqrt{mm}$)	4.2896
Error (%)	1.77

**Table 3:** J-integral comparison: exact method vs. FEM.

FEM mJ/mm^2^	0.01760
Exact mJ/mm^2^	0.01681
Error (%)	4.49

**Table 4:** Comparison of the $M^{e}$ and $\theta_{c}$ between the exact method and the FEM.

	$M^{e}$	$\theta_{c}$ (Degree)
Exact	0.32	−64.6
FEM	0.31	−64.5
Error (%)	3.12	0.15

## Data Availability

The original contributions presented in this study are included in the article. Further inquiries can be directed to the corresponding author.
